# The Role of Genetic Testing in Pediatric Expressive Language Delay: Evidence from the National Brain Gene Registry

**DOI:** 10.3390/genes17010061

**Published:** 2026-01-05

**Authors:** Shivani Waghmare, Alexa M. Taylor, Cecilia Bouska, Ana Moreno Chaza, Andrea Gropman

**Affiliations:** 1 Howard University College of Medicine, Washington, DC 20001, USA; shivani.waghmare@bison.howard.edu; 2 Children’s National Medical Center, Washington, DC 20010, USA; ataylor4@childrensnational.org (A.M.T.); cbouska@childrensnational.org (C.B.); amorenocha@childrensnational.org (A.M.C.); 3 St. Jude’s Children’s Research Hospital, Memphis, TN 38105-3678, USA

**Keywords:** expressive language disorder, genetic testing, brain gene registry, genetic variants, neurodevelopmental delay

## Abstract

Background/Objectives: Speech and language delay (SLD) is one of the most prevalent developmental conditions in childhood, with post-pandemic data indicating a notable increase in identified cases. Within this group, expressive language disorder (ELD) frequently appears alongside neurodevelopmental disorders such as autism spectrum disorder (ASD), epilepsy, and intellectual disability. Although awareness of ELD has grown, the role of genetic testing in its evaluation remains unclear, as such testing is not routinely pursued for isolated expressive language concerns. This gap highlights the need to better define the diagnostic value of genetic analysis and to examine the interval between an ELD diagnosis and the return of genetic testing results. Methods: This study investigated genetic contributions to ELD using the National Brain Gene Registry (BGR), a multisite database of rare neurodevelopmental disorders. Participants with ICD-10 code F80.1 were identified through electronic health records; demographic data, comorbidities, genetic variants, inheritance patterns, age at diagnosis, and timing of interventions were analyzed. Results: Of 687 BGR participants, 32 (4.7%) had documented ELD. The cohort, aged 3–19 years, presented with common comorbidities like developmental delays, ASD, epilepsy, and hypotonia. Across 42 genes, 49 unique variants were identified: 26 pathogenic or likely pathogenic, 22 variants of uncertain significance, and one benign variant. Seventeen variants were de novo, and 10 participants carried multiple variants. Most children (80%) received an expressive language diagnosis prior to genetic testing, with reports returned an average of 1.5 years following the diagnosis. Conclusions: Overall, children with ELD commonly carry genetic variants and neurodevelopmental comorbidities, yet genetic testing is typically pursued well after diagnosis and does not currently alter early management. These findings underscore the need for clearer, evidence-based guidelines to define when genetic testing adds diagnostic or prognostic value in the evaluation of ELD.

## 1. Introduction

Speech and language delay (SLD) is one of the most common developmental disabilities in childhood. In the United States, nearly one in twelve children aged 3–17 years was diagnosed with a disorder related to voice, speech, language, or swallowing [1]. Post-pandemic data shows that diagnoses of speech and language delay increased by 29% in 2021 and 84% in 2022 [2]. Compared to typically developing peers, children with speech and language delay or impairment exhibited significantly higher severity of emotional, behavioral, and Attention Deficit/Hyperactivity Disorder (ADHD) symptoms and are more likely to reach clinical thresholds for these conditions. The rising incidence shows the importance of closely examining and understanding the multifactorial influences of speech and language disorders.

Language and speech, while closely related, are distinct in their underlying neurocognitive mechanisms and developmental trajectories. Language is the use of arbitrary but socially agreed-upon symbols in a rule-governed system to convey meaning and enable creative expression beyond the present to discuss past or future events and abstract, hypothetical, or imaginary ideas. In contrast, speech refers to the articulation of the language. Language is categorized into two domains: receptive and expressive language. Receptive language is the ability to understand linguistic input, and expressive language is the ability to produce linguistic output [3].

Although these domains are distinct, language disorders are classified under two categories: expressive language disorder (ELD) (ICD-10 F80.1) and mixed expressive-receptive language disorder (ICD-10 F80.2). Receptive language disorder cannot be diagnosed without concurrent symptoms of expressive language impairments [4]. Expressive language development is a gradual process influenced by biological, social, and environmental factors. By 10–12 months of age, children typically begin to understand and produce their first words along with deictic and representational gestures, marking the development of expressive language. Early word comprehension and gesture use are closely linked and predict expressive vocabulary at 24 months. Between 20 and 24 months, expressive vocabulary expands further, and children begin forming two-word phrases. By age three, most have developed a relatively extensive mental lexicon, and their increasingly complex and grammatically accurate speech becomes intelligible to listeners beyond their immediate family [5].

ELD can contribute to unexpressed emotions and exacerbate some co-occurring neurodevelopmental conditions, like higher total disruptive behavior disorder symptoms, greater severity of ADHD symptoms, and increased hyperactive–impulsive traits. Expressive language disorders tend to develop later compared to receptive language disorders, as expressive language disorders are more strongly influenced by social and environmental factors. This increases the need to examine expressive language disorder through multiple lenses, including genetic, social, developmental, and environmental [6].

Several genes have been implicated in ELD, including *FOXP2* [7], *GRIN2A* [8], *UNC80* [9]. Additionally, ELD is a common feature of several neurogenetic syndromes, like MN1 C-terminal truncation (MCTT) syndrome [10], 22q11.2 deletion syndrome (22q11DS) [11], Pitt-Hopkins Syndrome [12], and Autism Spectrum Disorder (ASD) [13]. Genetic testing in relation to specific speech disorders, such as childhood apraxia of speech (CAS) has been well studied, where about 20 genes have been identified in association of CAS [14]. While these genetic discoveries support the clinical utility of genetic testing for CAS, there is a gap in research specifically addressing the genetic basis of expressive language disorder. Given this limited genetic research, the present study focuses on individuals with a documented diagnosis of ELD. Genetic testing is not currently standardized in the diagnostic evaluation of language disorders yet understanding the significance of genetic testing and its potential contribution for early and accurate diagnoses is essential.

The purpose of this study is to determine whether genetic testing can help identify genetic variants associated with ELD. This is accomplished by using the data in the Brain Gene Registry Database, which collects data about the genes implicated in the various brain developmental conditions [15]. Since ELD diagnoses are often secondary to other neurodevelopmental disorders, early identification of underlying genetic etiologies may facilitate prediction of comorbid symptoms and guide timely interventions. For example, early detection of a specific neurodevelopmental disorder could allow providers to anticipate language difficulties and implement targeted interventions sooner.

## 2. Materials and Methods

### 2.1. Brain Gene Registry

The National Brain Gene Registry (BGR) is a collaborative source of data collection designed to further explore rare genetic variants involved in brain development [15]. Focusing on genes related to intellectual and developmental disabilities, 13 sites in the U.S. have recruited participants for this initiative. The BGR is a collaboration between Intellectual and Developmental Disabilities Research Centers (IDDRCs) across the United States, funded by the NIH National Center for Advancing Translational Sciences (NCATS) (CTSA grant number 1U01TR002764-01A). Recruitment is focused on individuals with variants affecting a single gene, rather than multigene deletions and duplications to allow for BGR data to assist in ClinGen’s gene-disease validity curation. Data is stored on the Collaborative Informatics Environment for Learning on Health Outcomes (CIELO) platform, which securely supports protected health information (PHI). The BGR collects data using the Rapid Neurobehavioral Assessment Protocol (RNAP), electronic health record (EHR), and ClinGen Genome Connect health surveys. The RNAP includes parent questionnaires, like the Vineland and Developmental Profile-4, along with a telehealth assessment performed by trained study staff [15]. A basic neurological assessment, rapid dysmorphology exam, digital block-style cognitive test, and autism assessment are completed during the telehealth [15]. EHR data is extracted based on institutional policies. Details like demographics, diagnosis codes, medications, encounters, imaging, and clinical notes are included in the search at Children’s National.

### 2.2. Data Collection and Analysis

Eligible participants were identified by querying the CIELO database for the ICD-10 code F80.1 for ELD, within the EHR data. From these records, we extracted data on demographics, variant classifications, inheritance patterns, ASD diagnoses and comorbidities, testing sample and report dates and age at SLD diagnosis and interventions. Using the EHR data, we collected dates of early intervention initiation, ELD diagnosis, and genetic report disclosure which allowed us to calculate the diagnostic timeline for each participant. We used descriptive statistics to summarize our data, as seen below.

## 3. Results

### 3.1. Participants

As of 7 August 2025, a total of 687 participants were enrolled in the BGR, of whom 32 (4.7%) had a documented diagnosis of ELD (ICD-10 code F80.1). Participant ages ranged from 3 to 19 years old (M = 10). This cohort included 18 males and 14 females. Self-reported race and ethnicity were as follows: 25 participants identified as White, 2 as Black, 1 as Asian, 1 as Native American, 1 as multiracial, and 2 did not disclose race. Reported ethnicities included 4 Hispanic/Latino, 18 non-Hispanic/Latino, and 10 undisclosed. A diagnosis of ASD was present in 14 of the participants. Commonly reported comorbidities included developmental delays (*n* = 19), epilepsy (*n* = 13), sleep disturbances (*n* = 6), and hypotonia (*n* = 7). The participant demographics are illustrated in Table 1.

### 3.2. Genetic Findings

**Figure 1:**
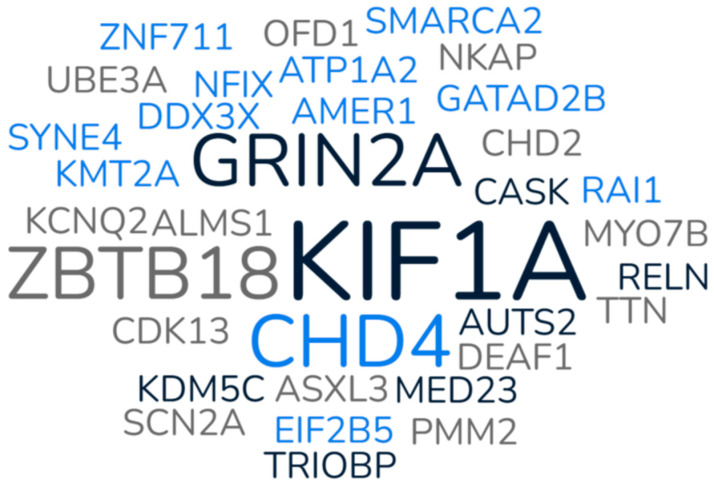
Frequencies of genes represented among participants. All genes were represented once except for *KIF1A* (*n* = 3), *GRIN2A* (*n* = 2), *ZBTB18* (*n* = 2), and *CHD4* (*n* = 2).

In this cohort, genetic variants were detected across 42 distinct genes (Figure 1) representing a total of 49 unique variants overall. The genetic analyses include 19 variants found by a multi-gene panel, 29 by exome sequencing (ES), and 1 (*RNU4-2*) by research genome which has not been confirmed in a CLIA-certified lab. Of these 49 variants, 26 were classified as pathogenic (P) or likely pathogenic (LP), 22 as variants of uncertain significance (VUS), and one as a benign polymorphism. Genetic analyses identified 17 occurring as de novo, 12 as inherited, and 20 as having an unknown inheritance pattern, based on the availability of parental testing. Additionally, 10 participants carried variants in more than one gene. All these variants were classified using the ACMG guidelines for classifying genetic variants.

### 3.3. Diagnosis to Treatment Timeline

**Figure 2:**
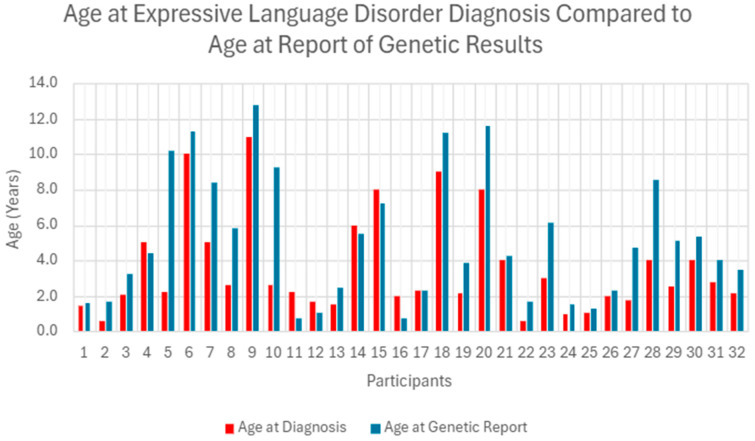
The participant’s age at diagnosis of ELD was compared to their age when they received the genetic report results.

The mean age at ELD diagnosis was 3.6 years old, with females averaging 3.2 years old and males averaging 3.8 years old. The mean age at initiation of speech and language intervention was 2.6 years old. Among the 14 participants (44%) who began receiving interventions after receiving a diagnosis, the average time to initiate services was 9.5 months. The remaining 18 participants (56%) began speech and language interventions an average of 2.3 years prior to receiving a formal diagnosis. Participants received genetic testing reports about 1.5 years after diagnosis and, on average, 2.5 years after initiating interventions. Genetic testing performed prior to a documented ELD diagnosis occurred in eight of the cases (25%) as represented by Table 2. Across these cases, four pathogenic, three likely pathogenic and two VUSs were seen in eight different genes. Figure 2 represents the graphical representation of comparison of age of diagnoses with age of genetic testing of participants in the study.

## 4. Discussion

Out of our study cohort, 75% of participants received ELD diagnoses before the initiation of genetic testing and reporting of results. This reflects a clinical reality in which management decisions and referrals for language-related disorders are often guided only by the child’s immediate functional needs rather than by a confirmed genetic diagnosis. Previous studies in speech-language pathology have similarly reported limited diagnostic yield for genetic testing conducted solely to determine the etiology of expressive language delay. For example, in one study a cohort of 127 children with developmental language disorder (DLD) were assessed by speech-language pathologists and geneticists and approximately 27% received a causative genetic diagnosis via Single Nucleotide Polymorphisms (SNP) arrays, gene panels, or ES. [16]. The average delay between initial clinical assessment and receipt of a genetic result was 10–12 months, underscoring the delay in integrating genetics into standard clinical workflows. Notably, clinical features such as motor delay, dysmorphic traits, or lower IQ were not significantly associated with genetic findings, suggesting that phenotype alone may not reliably guide referral for genetic evaluation.

It is important to note that exome sequencing did not become widely accessible until 2013–2014, with clinical genome sequencing emerging shortly thereafter, and early child neurology cohorts from that era demonstrated clear diagnostic benefits of genetic testing for conditions such as intellectual disability, autism spectrum disorder, and epilepsy—disorders that, much like in our cohort, often accompany severe expressive language impairments [17]. However, many individuals in our study exhibited language delay before these advanced sequencing technologies were clinically available, limiting opportunities for earlier identification of underlying etiologies. Although research specifically focused on expressive language disorder remains limited, studies in related language disorders have shown meaningful diagnostic yield from genetic evaluation. Current American Academy Pediatrics (AAP) and American Academy of Family Physicians (AAFP) guidelines recommend considering genetic testing when no clear cause is identified after a comprehensive history and physical examination, particularly in cases of severe language impairment, co-occurring neurodevelopmental conditions, or a suggestive family history [18,19]. Recent evidence further supports routine molecular testing for severe developmental language disorder, with clinically significant copy number variants identified in roughly 7% of affected children and whole exome sequencing yielding diagnoses in approximately 7.5% [20,21]. Despite these advances, there remains a lack of robust data guiding when clinicians should pursue genetic workups for isolated expressive language disorder, underscoring the need for evidence-based recommendations to inform evaluation practices for this population.

Another important point to note is that in our study around 56% of patients started receiving therapeutic interventions even before the formal diagnoses of ELD. This underscores the importance of the broad developmental screeners, such as the Ages and Stages Questionnaire (ASQ), which can identify delays across multiple developmental domains, prompting more targeted follow-up assessments, like the Language Development Survey or the Early Language Scale [22]. Evidence synthesis shows several parent-reported expressive language tools achieve high sensitivity (~88–93%) and specificity (~85–88%) for detecting expressive delay [23]. Incorporating these tools into early developmental evaluations can help guide timely interventions even in the absence of genetic evaluations.

The interventions for ELD typically begin with a formal audiological evaluation, followed by intensive, goal-directed therapy targeting expressive deficits (e.g., vocabulary development, syntax, motor-planning for CAS). Therapy may be delivered in clinic, at school, in the home, or via telehealth with intensity and duration tailored to severity of the disorder and functional goals [24]. Early intervention targets heightened neuroplasticity during early childhood, when neural pathways involving speech, language, and executive function are most malleable [25].

Developmental neuroscience syntheses emphasize that earlier, enriched language input and caregiver-mediated strategies capitalize on critical and sensitive periods to strengthen Perisylvian and Frontostriatal circuits [25]. This mitigates cascading effects on cognition and behavior, delayed intervention risks consolidation of inefficient networks, and broader neurodevelopmental sequelae [25]. Thus, early intervention should be initiated based on developmental delays themselves rather than waiting for genetic testing results, since eligibility for services is determined by a 25% delay in any developmental domain, including communication.

## 5. Limitations and Future Directions

This study focused on a single diagnostic category of ELD, which limits the generalizability of our findings across the broader spectrum of speech and language impairments. Additionally, most participants underwent genetic testing as part of an expanded neurodevelopmental evaluation, introducing potential surveillance bias. As a result, individuals with isolated ELD were underrepresented.

Another key limitation of this study is the use of the Brain Gene Registry, which predominantly includes individuals with established neurodevelopmental diagnoses. As a result, the cohort includes more severe or syndromic presentations, which may limit the generalizability of our findings to children with isolated expressive language delay in the general pediatric population. Future research should incorporate broader inclusion criteria, including receptive, expressive, and mixed language disorders and children who have not yet undergone genetic testing. Such an approach may reduce selection bias and clarify the important diagnostic value of genetic evaluation in these populations. Expanding recruitment across diverse populations and clinical settings, along with long-term follow-up data, will strengthen our understanding of the role of genetics in pediatric language disorders and inform evidence-based testing guidelines.

## Figures and Tables

**Table 1:** Participant demographics. U = unknown; P = pathogenic; LP = likely pathogenic; VUS = variant of uncertain significance; B = benign; autosomal dominant; AR = autosomal recessive.

ID	Age	Gender	Race	Ethnicity	Gene (Classification)	Inheritance Pattern
1	6	F	White	U	*ZBTB18* (P)	De novo
					*PMM2* (VUS)	Maternal
2	8	F	White	Hispanic/Latino	*MED23* (LP)	De novo
					*TTN* (VUS) *	Maternal
					*TTN* (VUS) *	Paternal
					*MYO7B* (VUS) *	Paternal
					*MYO7B* (VUS) *	Maternal
3	5	M	White	U	*GATAD2B* (P)	Unknown
					*EIF2B5* (LP)	Unknown
4	11	F	White	Not Hispanic or Latino	*CHD4* (LP)	De novo
					*KCNQ2* (P)	Paternal
5	14	M	White	Not Hispanic or Latino	*ZBTB18* (LP)	De novo
6	16	F	White	Not Hispanic or Latino	*KDM5C* (P)	De novo
					*ALMS1* (VUS)	Maternal
					*SYNE4* (VUS)	Paternal
					*TRIOBP* (VUS)	Maternal
7	14	M	White	Not Hispanic or Latino	*NKAP* (VUS)	Maternal
8	7	F	White	Hispanic/Latino/Spanish Origin	*CASK* (LP)	De novo
9	19	M	White	Not Hispanic or Latino	*AMER1* (VUS)	Unknown
					*AUTS2* (VUS)	Unknown
10	15	M	White	Not Hispanic or Latino	*KMT2A* (P)	Unknown
11	4	F	White	Not Hispanic or Latino	*NFIX* (LP)	De novo
12	8	M	White	Not Hispanic or Latino	*ATP1A2* (P)	Unknown
13	7	M	White	Not Hispanic or Latino	*CHD2* (LP)	De novo
14	12	F	White	U	*GRIN2A* (VUS)	Unknown
					*SCN2A* (LP)	Unknown
15	11	M	Other	U	*GRIN2A* (VUS)	Unknown
					*RELN* (B)	Unknown
16	3	M	White	U	*KIF1A* (VUS)	Unknown
17	5	F	White	Not Hispanic or Latino	*KIF1A* (P)	De novo
18	15	M	White	Not Hispanic or Latino	*KIF1A* (VUS)	Unknown
					*RAI1* (VUS)	Unknown
					*SMARCA2* (VUS)	Unknown
19	7	M	Unknown	U	*CDK13* (VUS)	Unknown
20	14	M	White	Hispanic/Latino	*ZNF711* (P)	Maternal
21	15	F	Multiracial	Not Hispanic or Latino	*DEAF1* (P)	De novo
22	7	M	White	U	*OFD1* (P)	Unknown
23	10	F	White	U	*DDX3X* (P)	De novo
24	6	M	White	U	*ASXL3* (P)	De novo
25	3	F	South Asian/White	U	*UBE3A* (P)	Unknown
26	4	M	White	Not Hispanic or Latino	*ZBTB18* (P)	De novo
27	12	F	Black	Not Hispanic or Latino	*SHANK3* (VUS)	Unknown
					*ARID1A* (VUS)	Unknown
					*TFE3* (VUS)	De novo
28	12	M	Native American	Hispanic	*SETD5* (VUS)	Unknown
29	6	F	White	Not Hispanic or Latino	*RNU4-2* (P)	De novo
30	11	M	White	Not Hispanic or Latino	*OPHN1* (LP)	De novo
31	8	F	Black	Not Hispanic or Latino	*SLC6A1* (LP)	De novo
32	9	M	White	Not Hispanic or Latino	*CACNA1A* (P)	Maternal

* Participant 2 had two unique variants in *TTN* and *MYO7B*.

**Table 2:** Participants (*n* = 8) that received genetic testing prior to an ELD e diagnosis.

ID	Gender	Race	Gene	Variant	Classification	Inheritance	Age at Report (Years)	Age at Dx (Years)
1	F	White	ZBTB18	c.1484G > A p.R495H	P	De novo	1.6	1.8
4	F	White	CHD4	c.4147G > T g.6692192C > A IVS27+1G > T	LP	De novo	4	5
			KCNQ2	c.553G > A p.A185T	P (reclassified from LP)	AD		
11	F	White	NFIX	c.361A > G p.Lys121Glu	LP	De novo	0.75	2.25
12	M	White	ATP1A2	c.2936C > T; p.Pro979Leu	P	Unknown	1	1.6
14	F	White	GRIN2A	c.13G > A; p.Gly5Ser	VUS	Unknown	5.5	6
			SCN2A	c.3955C > T;p. Arg1319Trp	LP	Unknown		
16	M	White	KIF1A	c.4721C > T; p.Pro1574Leu	VUS	Unknown	7.2	8
17	F	White	KIF1A	c.647G > A; p.Arg216His	P	De novo	0.7	2
21	F	Multiracial	DEAF1	c.762A > C p.R254S	P	De novo	2.25	2.3

## Data Availability

The data presented in this study is available on request from the corresponding author due to linkage of the data with protected health information.

## References

[1] (2024). Quick Statistics About Voice, Speech, Language|NIDCD. https://www.nidcd.nih.gov/health/statistics/quick-statistics-voice-speech-language.

[2] Speech_Pathology_Research_Brief.pdf. https://www.komodohealth.com/perspectives/louder-than-words-pediatric-speech-disorders-skyrocket-throughout-pandemic/.

[3] Feldman H.M. (2019). How young children learn language and speech. Pediatr. Rev..

[4] Leonard L.B. (2009). Is expressive language disorder an accurate diagnostic category?. Am. J. Speech Lang. Pathol..

[5] Sansavini A., Favilla M.E., Guasti M.T., Marini A., Millepiedi S., Di Martino M.V., Vecchi S., Battajon N., Bertolo L., Capirci O. (2021). Developmental language disorder: Early predictors, age for the diagnosis, and diagnostic tools. A scoping review. Brain Sci..

[6] Gremillion M.L., Martel M.M. (2014). Merely misunderstood? Receptive, expressive, and pragmatic language in young children with disruptive behavior disorders. J. Clin. Child. Adolesc. Psychol..

[7] Morgan A., Fisher S.E., Scheffer I., Hildebrand M. (2016). *FOXP2*-related speech and language disorder. GeneReviews® [Internet].

[8] Strehlow V., Myers K.A., Morgan A.T., Scheffer I.E., Lemke J.R., Adam M.P., Feldman J., Mirzaa G.M., Pagon R.A., Wallace S.E., Amemiya A. (2016). *GRIN2A*-related disorders. GeneReviews® [Internet].

[9] Bramswig N.C., Zaki M.S., Adam M.P., Feldman J., Mirzaa G.M., Pagon R.A., Wallace S.E., Amemiya A. (2017). UNC80 Deficiency. GeneReviews® [Internet].

[10] Mak C.C.Y., Fung J.L.F., Lee M., Lin A.E., Amiel J., Doherty D., Gordon C.T., Chung B.H.Y., Adam M.P., Feldman J., Mirzaa G.M., Pagon R.A., Wallace S.E., Amemiya A. (2020). *MN1* C-terminal truncation syndrome. GeneReviews® [Internet].

[11] Everaert E., Selten I., Boerma T., Houben M., Vorstman J., de Wilde H., Derksen D., Haverkamp S., Wijnen F., Gerrits E. (2023). The language profile of preschool children with 22q11.2 deletion syndrome and the relationship with speech intelligibility. Am. J. Speech Lang. Pathol..

[12] Sweetser D.A., Gipson K.S., Adam M.P., Feldman J., Mirzaa G.M., Pagon R.A., Wallace S.E., Amemiya A. (2012). Zar-Kessler CPitt-Hopkins Syndrome. GeneReviews® [Internet].

[13] McDaniel J., D’Ambrose Slaboch K., Yoder P. (2018). A meta-analysis of the association between vocalizations and expressive language in children with autism spectrum disorder. Res. Dev. Disabil..

[14] Kaspi A., Hildebrand M.S., Jackson V.E., Braden R., van Reyk O., Howell T., Debono S., Lauretta M., Morison L., Coleman M.J. (2023). Correction: Genetic aetiologies for childhood speech disorder: Novel pathways co-expressed during brain development. Mol. Psychiatry.

[15] Baldridge D., Kaster L., Sancimino C., Srivastava S., Molholm S., Gupta A., Oh I., Lanzotti V., Grewal D., Riggs E.R. (2024). The Brain Gene Registry: A data snapshot. J. Neurodev. Disord..

[16] Plug M.B., van Wijngaarden V., de Wilde H., van Binsbergen E., Stegeman I., van den Boogaard M.H., Smit A.L. (2021). Clinical characteristics and genetic etiology of children with developmental language disorder. Front. Pediatr..

[17] Korf B.R., Rehm H.L. (2013). New approaches to molecular diagnosis. JAMA.

[18] Koopman J.J., Fiore D.C., Thiele K. (2025). Approach to Developmental Screening and Surveillance in Young Children. Am. Fam. Physician..

[19] Rodan L.H., Stoler J., Chen E., Geleske T., Council on Genetics (2025). Genetic Evaluation of the Child with Intellectual Disability or Global Developmental Delay: Clinical Report. Pediatrics.

[20] Kalnak N., Stamouli S., Peyrard-Janvid M., Rabkina I., Becker M., Klingberg T., Kere J., Forssberg H., Tammimies K. (2018). Enrichment of rare copy number variation in children with developmental language disorder. Clin. Genet..

[21] Yahia A., Li D., Lejerkrans S., Rajagopalan S., Kalnak N., Tammimies K. (2024). Whole exome sequencing and polygenic assessment of a Swedish cohort with severe developmental language disorder. Hum. Genet..

[22] Srivastava S., Cohen J.S., Vernon H., Barañano K., McClellan R., Jamal L., Naidu S., Fatemi A. (2014). Clinical whole exome sequencing inchild neurology practice. Ann. Neurol..

[23] Feltner C., Wallace I.F., Nowell S.W., Orr C.J., Raffa B., Middleton J.C., Vaughan J., Baker C., Chou R., Kahwati L. (2024). Screening for speech and language delay and disorders in children 5 years or younger: Evidence report and systematic review for the US preventive services task force. JAMA.

[24] Barry M.J., Nicholson W.K., Silverstein M., Chelmow D., Coker T.R., Davis E.M., Donahue K.E., Jaén C.R., Li L., US Preventive Services Task Force (2024). Screening for speech and language delay and disorders in children: US prev33entive services task force recommendation statement. JAMA.

[25] Nelson C.A., Sullivan E., Engelstad A.M. (2024). Annual Research Review: Early intervention viewed through the lens of developmental neuroscience. J. Child. Psychol. Psychiatry.

